# Effect of land cover and landscape fragmentation on anopheline mosquito abundance and diversity in an important Colombian malaria endemic region

**DOI:** 10.1371/journal.pone.0240207

**Published:** 2020-10-15

**Authors:** Juan C. Hernández-Valencia, Daniel S. Rincón, Alba Marín, Nelson Naranjo-Díaz, Margarita M. Correa

**Affiliations:** 1 Grupo de Microbiología Molecular, Escuela de Microbiología, Universidad de Antioquia, Medellín, Colombia; 2 Universidad de Antioquia, Medellín, Colombia; Faculty of Science, Ain Shams University (ASU), EGYPT

## Abstract

Landscape structure influences the distribution and abundance of anopheline mosquitoes and has an indirect impact on malaria transmission. This work aimed to determine the effect of land cover and landscape fragmentation on anopheline mosquito abundance and diversity in an important Colombian malaria endemic area, the Bajo Cauca region. Diversity indices were calculated for *Anopheles* mosquitoes collected in various localities of the region. Land cover types were characterized using orthorectified aerial photographs to estimate landscape metrics. The relationship between landscape fragmentation and species diversity was evaluated by regression analysis. The correlation between species abundance and land cover types was determined using canonical correspondence analyses. Results showed a statistically significant tendency for a lower diversity of the *Anopheles* community in landscapes with higher patch number, patch density and effective mesh size. For most species, there was evidence of a significant relationship between species abundance and land covers modified by anthropic activities which generate forest loss. These results indicate that activities that modify the landscape structure and land cover composition generate changes that affect the spatial distribution and composition of epidemiologically-important *Anophele*s species, which may impact malaria distribution in a region. This information is useful to guide control interventions that promote unfavorable landscapes for malaria vector propagation.

## Introduction

Malaria is a problem of public health in Colombia. In 2018, the country occupied the third position in the number of cases among the countries in Latin America [1], with 61,200 cases registered [2]; however, underestimation is presumed, with numerous cases not being reported to the surveillance system [3]. The Bajo Cauca region in NW Colombia, where this study was conducted, has historically registered among the highest number of malaria cases of the total in the country [4].

Landscape structure determination includes the description of the spatial pattern of elements (land covers) and their connections [5]; a relevant aspect for the organisms inhabiting a landscape, because land covers give the physical appearance to earth and ultimately influence their biology [6, 7]. Mosquito spatial distribution is influenced by abiotic factors such as precipitation and temperature, and biotic features that include among others, host, vegetation conditions and land covers; they determine breeding site availability, the physical environment of the adult mosquito [7–13], and influence mosquito-host presences [11]. Studies that have estimated the relationship between environmental variables and *Anopheles* species, indicate that land cover composition is one of the most influential factors affecting species abundance and distribution [7, 14–16]. Furthermore, human activities that modify land cover composition impact the dynamics of disease transmission [17]. Regarding mosquitoes, landscape anthropization has often resulted in anthropophilic species proliferation, which in general have greater epidemiological importance [18–20]. Few studies have evaluate the association of land cover types and mosquito biology, and specifically, the impact of land cover alterations in *Anopheles* composition, distribution and behavior, aspects known to affect malaria transmission [19, 21–24]. Results of a recent study conducted in the Colombian malaria endemic Urabá region showed that land covers derived from anthropic activities favored the presence and abundance of the main malaria vectors [16]. Also, in northern Peruvian Amazon, deforestation was associated with increased human-biting activity by the primary malaria vector *Anopheles darlingi* [22], and with larval habitat availability which increased vector presence [23]. Similarly, deforestation and changes in land cover were linked to an increase in the reproduction rate and vectorial capacity of the main African malaria vectors *Anopheles gambiae* and *Anopheles funestus* [25].

In the Colombian malaria endemic Bajo Cauca region, mining, livestock and farming are the main economic activities [26, 27]; these anthropic activities are known to significantly alter land cover and landscape composition, modifying the environmental conditions that affect malaria incidence [17, 28, 29]. Previous studies in this region have been mainly directed to identify *Anopheles* species composition, natural infection, and behavior [30, 31], genetic population structure and phylogeny [32, 33]; however, the relationship between landscape structure and the *Anopheles* community has not yet been established. Therefore, this study was conducted to test the hypothesis that in the Bajo Cauca region, *Anopheles* species abundance is related to land covers derived from anthropic activities and that species diversity is influenced by landscape fragmentation. This information will contribute to the understanding of malaria transmission dynamics; in addition, it provides the bases for control interventions that include epidemiologically responsible landscape management for malaria prevention.

## Materials and methods

### Mosquito collection and identification

**Fig 1:**
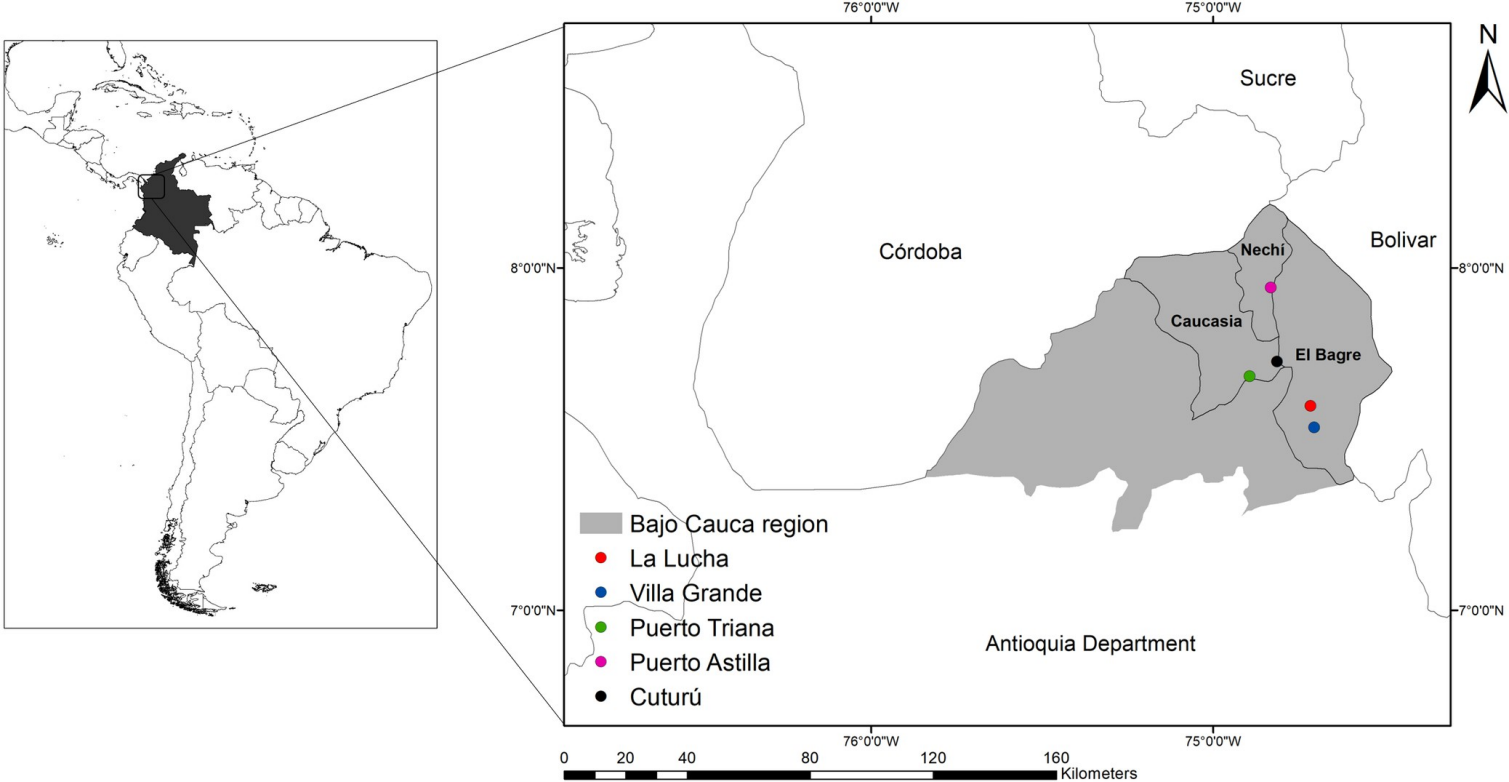
Localities for mosquito collection in the Bajo Cauca region. Villa Grande and La Lucha in El Bagre (BAG) municipality, Cuturú and Puerto Triana in Caucasia (CAU), and Puerto Astilla in Nechí (NEC).

*Anopheles* mosquitoes were collected in the malaria endemic Bajo Cauca region in Antioquia Department, Colombia (Fig 1). The Bajo Cauca region is part of the Magdalena-Urabá Moist Forest ecoregion [34]. The main economic activities in this region include open-pit mining, livestock, agriculture, pisciculture and logging [26, 27]. Collections were performed in five localities during three consecutive nights, sampling two different sites each night, for a total of six sampling sites at each locality. The mosquitoes were collected by protected human-landing catches (HLC), under an informed consent agreement and protocol reviewed and approved by Comité de Bioetica de la Facultad Nacional de Salud Pública, UdeA, Acta 063–2013. Collections were done indoors and outdoors (within ~10 meters of the house), from 18:00 to 24:00 h. Some mosquitoes were also collected resting in livestock corrals. The specimens were morphologically identified using a taxonomic key [35]. After DNA extraction [36], the species assignation was confirmed by Polymerase Chain Reaction—Restriction Fragment Length Polymorphisms (PCR-RFLP) of the ITS2 region [37, 38].

### Landscape analysis

Coordinates corresponding to collection sites were registered using a global positioning system (Garmin MAP76 CSX®). An area of 1.5 Km of radius from the collection site was characterized; this distance corresponds to the maximum average dispersion range reported for *Anopheles* [39–41]. The land cover classification was performed on orthorectified aerial photographs (scale 1:10,000) supplied by the “Secretaria de Planeación” of Antioquia Department, taken in the last quarter of 2015. During orthorectification, geometric and scale distortions such as topographic variations and earth’s curvature were eliminated. Land cover types were characterized in the orthophotos by visual inspection with ArcGIS 10.2 [42] and labeled according to categories of national land cover legends by the Instituto de Hidrología, Meteorología y Estudios Ambientales (IDEAM) of the Colombian government [43] (S1 Table). Land uses were determined according to aerial photographs and field observations at the localities. The maximum period between mosquito collection and aerial photographs was under 14 months. Estimated landscape indices included, the total landscape area, number of classes or covers, patch area, total cover area, percentage cover area (PA), and mean patch size (MPS). Measures of landscape fragmentation comprised, the number of patches (NP), number of fragments per unit area or patch density (PD), and the effective mesh size (MSIZ). Additionally, landscape diversity was estimated using Shannon’s diversity index (SHDI), calculated with the number of land covers and area uniformity among the cover types [44]. All landscape indices were obtained in the V-LATE 2.0 software [45].

### Data analysis

Species accumulation curves for the *Anopheles* community were carried out with the software EstimateS V. 9.1.0 [46]. Estimates of *Anopheles* diversity included, the number of collected specimens, species richness (S), Shannon-Weaver index (H’), Simpson (1-D), Equitability (J), and Dominance (D). Simple linear regression was used to evaluate the relationship between *Anopheles* diversity (Shannon-Weaver index) with landscape fragmentation indices and landscape diversity, using the PAST software v. 3.15 [47].

The relationships between *Anopheles* species abundance and land cover types were determined for the entire region by Canonical Correspondence Analysis (CCA); this multivariate gradient analysis is widely used to relate species with environmental variables [48]. For this analysis, a 400-meter radius area was considered which corresponds to the commonly reported flying range for *Anopheles* [39, 40]. To avoid bias, the sites with overlapping areas were excluded; thus, only 15 collection points were included in the analysis. The CCA was performed using a matrix of species abundance and land cover area per collection site. To correct possible statistical errors associated with rare or dominant species, a logarithmic transformation was applied to the data matrix. Variance Inflation Factors (VIF) were calculated and indicated no collinearity among land cover variables (S1 Data). The statistical significance of the CCA model and canonical axes were evaluated by permutation tests. The model and its significance were estimated under the Vegan library in R Studio v. 3.4.1 [49, 50].

## Results

### *Anopheles* species abundance and diversity

A total of 2,458 *Anopheles* mosquitoes corresponding to 10 species were collected in six sampling sites of each of the five localities visited, during 180 hours of sampling. *Anopheles braziliensis* (n = 874, 35.6%), *An*. *nuneztovari* (n = 581, 23.6%) and *An*. *darlingi* (n = 495, 20.1%) were the most abundant species and were present in all localities, except *An*. *braziliensis* in La Lucha-BAG. Other species collected were *An*. *albitarsis* s.l. (10.6%), *An*. *triannulatus* s.l. (5.7%), *An*. *punctimacula* (2%), species near *An*. *peryassui* (1.3%) first described in this region [51], *An*. *oswaldoi* (0.8%), *An*. *rangeli* (0.1%) and *An*. *pseudopunctipennis* (0.04%) (Table 1). Species abundance did not show a normal distribution (*p<* 0.05), except for *An*. *darlingi* (W = 0.86 *p>* 0.05), *An*. *triannulatus* s.l. (W = 0.87; *p>* 0.05) and *An*. *albitarsis* s.l. (W = 0.91, *p>* 0.05).

**Table 1:** *Anopheles* species abundances in localities of the Bajo Cauca region, Colombia.

Municipality / Locality / Coordinates	Year and month of collection	Species	*n* (%) [HLC/RC]	Range of mosquitoes captured per night (Mean/SD)
**El Bagre**	2013 September	*An*. *nuneztovari*	537 (86.8) [413/124]	10–197 (89.5/±74.1)
La Lucha		*An*. *triannulatus* s.l.	46 (7.4) [34/12]	1–32 (7.5/ ±12.1)
N 7°35'43' W 74°42'56''				
		*An*. *darlingi*	34 (5.5) [34/0]	0–57 (5.7/±6.9)
		*An*. *albitarsis* s.l.	1 (0.2) [1/0]	0–2 (0.2/±0.4)
		*An*. *pseudopunctipennis*	1 (0.2) [1/0]	0–1 (0.2/±0.4)
Villa Grande	2013 September	*An*. *darlingi*	79 (67.5) [79/0]	0–57 (13.2/± 22.1)
N 7°32'0'' W 74°42'16''				
		*An*. *nuneztovari*	20 (17.1) [20/0]	0–8 (3.3/±3.4)
		*An*. *triannulatus* s.l.	15 (12.8) [15/0]	0–9 (2.5/±3.7)
		*An*. *albitarsis* s.l.	2 (1.7) [2/0]	0–2 (0.3/±0.8)
		*An*. *braziliensis*	1 (1.7) [1/0]	0–1 (0.2/±0.4)
**Nechí**	2013 September	*An*. *braziliensis*	763 (67.3) [763/0]	50–222 (127.2/±70.8)
Puerto Astilla				
N 7°56'31'' W 74°49'45''				
		*An*. *darlingi*	223 (19.7) [223/0]	5–143 (37.2/±53.1)
		*An*. *albitarsis* s.l.	58 (5.1) [58/0]	0–20 (9.7/±8.1)
		*An*. *punctimacula*	49 (4.3) [49/0]	0–45 (8.2/±18.1)
		near *An*. *peryassui* *	34 (3.0) [34/0]	1–12 (5.7/±3.8)
		*An*. *triannulatus* s.l.	5 (0.4) [5/0]	0–2 (0.8/± 0.7)
		*An*. *nuneztovari*	2 (0.2) [2/0]	0–2 (0.3/±0.8)
**Caucasia**	2014 May	*An*. *braziliensis*	106 (40.3) [84/22]	0–31 (17.6/±20.9)
Cuturú				
N 7°43'29'' W 74°47'12''				
		*An*. *albitarsis* s.l.	75 (28.5) [54/21]	0–74 (12.5/±30.1)
		*An*. *darlingi*	50 (19) [40/10]	0–31 (8.3/±11.3)
		*An*. *nuneztovari*	15 (5.7) [12/3]	0–14 (5.6 /±5.6)
		*An*. *triannulatus* s.l.	13 (4.9) [12/1]	0–10 (2.2/±3.9)
		*An*. *oswaldoi*	3 (1.1) [0/3]	0–3 (0.5/±1.2)
		*An*. *rangeli*	1 (0.4) [0/1]	0–1 (0.2/±0.4)
Puerto Triana	2014 May	*An*. *albitarsis* s.l.	125 (38.5) [21/104]	0–104 (20.8/±41.1)
N 7°40'58'' W 74°56'36''				
		*An*. *darlingi*	109 (33.5) (69/40)	4–46 (18.2/±14.7)
		*An*. *triannulatus* s.l.	61 (18.8) [19/42]	0–49 (10.2/±19.2)
		*An*. *oswaldoi*	16 (4.9) [1/15]	0–9 (2.7/±3.5)
		*An*. *nuneztovari*	7 (2.2) [0/7]	0–5 (1.2/±2.0)
		*An*. *braziliensis*	4 (1.2) (0/4)	0–3 (0.7/±1.2)
		*An*. *rangeli*	2 (0.6) [2/0]	0–2 (0.33/±0.8)
		*An*. *punctimacula*	1 (0.3) [1/0]	0–1 (0.2/±0.4)

***n*:** total number of specimens collected by locality. **HLC:** number of specimens collected by human-landing catches. **RC:** number of specimens collected resting in livestock corrals. **Mean/SD: Mean:** average of specimens per night. **DS:** standard deviation.

* Near *An*. *peryassui* first described in this region [51].

The accumulation curve for the Bajo Cauca region predicted ten species and reached a horizontal asymptote, indicating that the sampling effort was enough. At the local scale, although no locality reached the asymptote, the curves are seen to be close to reaching it, indicating that the sampling effort was acceptable (S1 Fig). In general, the *Anopheles* community showed low diversity (Shannon-Weaver H’< 2). The highest species richness was registered in Puerto Triana-CAU with eight species and the lowest in La Lucha-BAG and Villa Grande-BAG with five species each. The highest *Anopheles* diversity was found in Cuturú-CAU and Puerto Triana-CAU with a Shannon-Weaver index of 1.424 and 1.382, Simpson 0.71 and 0.70 and Equitability (J) of 0.73 and 0.66, respectively; in accordance, these localities also had the lowest Dominance (D) value with 0.29 and 0.30, respectively, which indicates the uniformity of the *Anopheles* community. The locality with the lowest species diversity was La Lucha-BAG (H’ = 0.49) that showed the highest Dominance D value (0.76), with *An*. *nuneztovari* as the dominant species (Table 2).

**Table 2:** *Anopheles* diversity indexes in the Bajo Cauca region by locality.

Diversity Indexes	El Bagre		Nechí	Caucasia		Bajo Cauca ^Ω^
	La Lucha	Villa Grande	Puerto Astilla	Cuturú	Puerto Triana	
**Species richness**	5ǂ	5ǂ	7	7	8*	10
**Abundance**	619	117	1134	263	325	2450
**Dominance D**	0.7612 ^-^	0.5019	0.4968	0.2857^+^	0.2987^+^	0.238
**Simpson 1-D**	0.2388 ^-^	0.4981	0.5032	0.7143^+^	0.7013^+^	0.762
**Shannon H’**	0.4966 ^-^	0.9407	1.014	1.424^+^	1.382^+^	1.620
**Equitability J**	0.3086 ^-^	0.5845	0.5213	0.7317^+^	0.6646^+^	0.7036
**Chao-1**	6	5	7	7	8	10

*: highest richness

ǂ: lowest richness

+: highest diversity

**-**: lowest diversity

Ω: index for Bajo Cauca region.

### Landscape structure description

**Fig 2:**
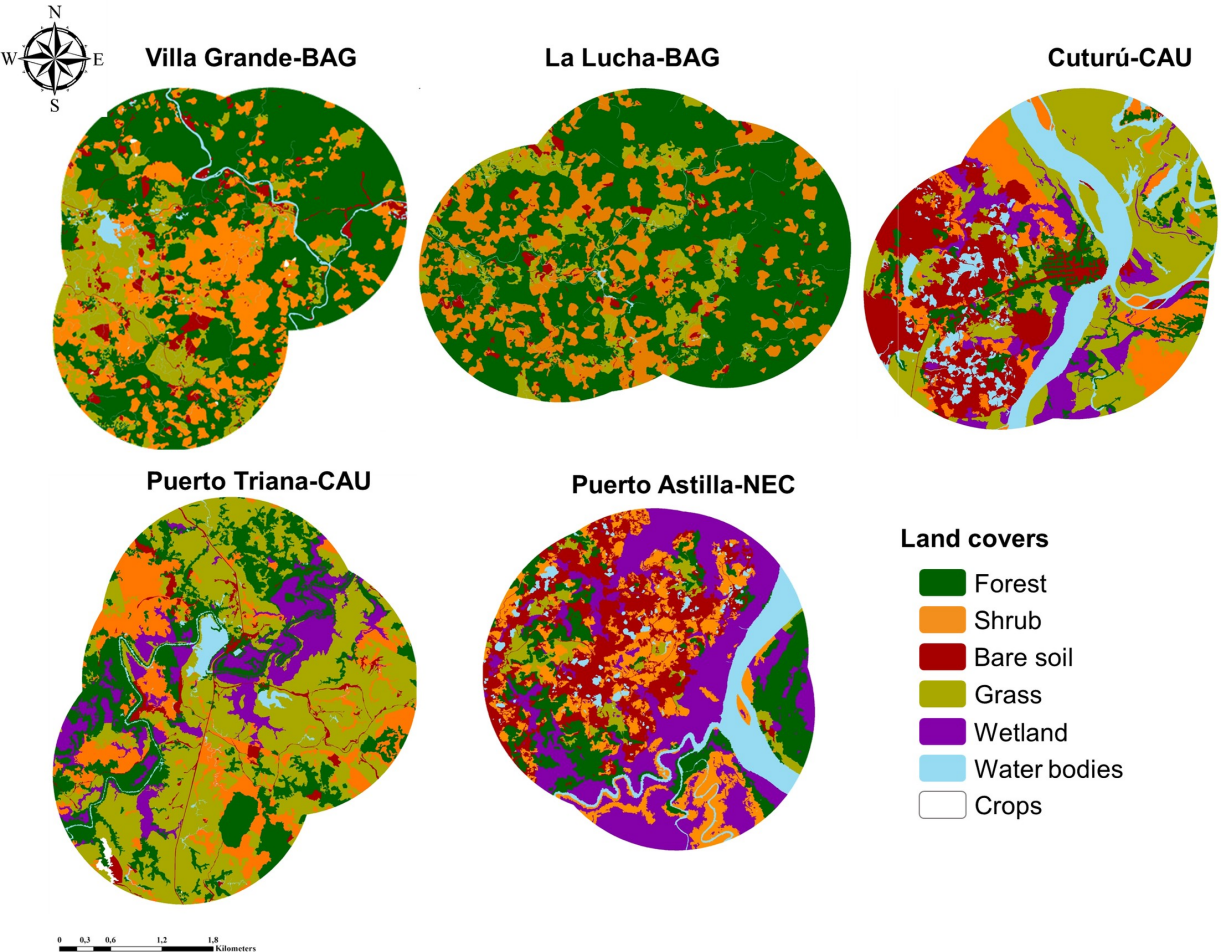
Land cover maps for the localities. Images of land cover types were obtained using orthorectified aerial photographs on an area of 1.5 Km of radius from the collection site.

A total of seven land cover types were detected and included, forest, water body, grass, shrub, bare soil, crop and wetland (Fig 2, Table 3). The area of cover types presented a normal distribution (*p>* 0.05), except for crop (W = 0.75; *p<* 0.05). La Lucha-BAG and Villa Grande-BAG had similar mean patch sizes (1.2 and 1.3 Ha, respectively); they also had similar land cover compositions, except for the presence of crop cover in Villa Grande-BAG (< 1Ha); forest was the matrix coverage defined for both localities. Cuturú-CAU, Puerto Triana-CAU, and Puerto Astilla-NEC presented landscapes without a defined matrix, as a result of the landscape interventions by mining activities. These localities also showed higher mean patch sizes and land cover types than those observed in La Lucha-BAG and Villa Grande-BAG (2.37, 2.45, and 1.5 Ha, respectively). A larger number of land cover types with uniformity of area indicate higher landscape diversity in the CAU and NEC localities (SHDI = 1.688, 1.416 and 1.670) (Table 3). In contrast, the BAG localities had lower landscape diversity (SHDI = 1.203 and 0.988) (Table 3).

**Table 3:** Land covers and landscape metrics for the localities of Bajo Cauca region.

Locality	Land cover	Metrics						
		Area (Ha)	NP	MPS (Ha)	PA (%)	PD	MSIZ (Ha)	SHDI
**La Lucha-BAG**	Forest	906.26	243	3.73	63.57	-	-	-
	Shrub	329.35	286	1.15	23.1			
	Grass	142.13	252	0.56	9.97			
	Bare soil	37.48	236	0.16	2.63			
	Water bodies	10.36	56	0.19	0.73			
	**Total**	1425.58	1073	1.33	-	0.83	149	0.988
**Villa Grande-BAG**	Forest	688	157	4.38	55.58	-	-	-
	Shrub	264	295	0.89	21.3			
	Grass	186	194	0.96	15			
	Bare soil	62	269	0.23	5.03			
	Water bodies	37	97	0.38	3			
	Crops	1	15	0.09	0.1			
	**Total**	1238	1027	1.2	-	0.75	62.14	1.203
**Puerto Astilla-NEC**	Wetland	314.18	94	3.34	30.31	-	-	-
	Bare soil	207.82	118	1.76	20.05			
	Shrub	185.77	234	0.79	17.92			
	Forest	169.74	84	2.02	16.38			
	Water bodies	111.41	140	0.8	10.75			
	Grass	47.56	30	1.59	4.59			
	**Total**	1036.47	700	1.5	-	0.68	73.33	1.67
**Cuturú-CAU**	Grass	360.6	57	6.33	31.79	-	-	-
	Bare soil	227.73	46	4.95	20.07			
	Water bodies	194.55	142	1.37	17.15			
	Shrub	142.4	62	2.3	12.55			
	Wetland	111.63	70	1.59	9.84			
	Forest	97.28	100	0.97	8.58			
	**Total**	1134.18	477	2.37	-	0.42	43.46	1.688
**Puerto Triana-CAU**	Grass	620	113	5.49	44.11	-	-	-
	Forest	328.16	133	2.47	23.35			
	Shrub	206.87	124	1.67	14.72			
	Wetland	191.19	89	2.15	13.6			
	Bare soil	45.53	67	0.68	3.24			
	Water bodies	10.08	44	0.23	0.72			
	Crops	3.73	3	1.24	0.27			
	**Total**	1405.56	573	2.45	-	0.41	23.39	1.416

**Ha:** hectares. **NP:** number of patches. **MPS:** mean patch size. **PA:** percentage cover area**. PD:** patch density. **MSIZ**: effective mesh size. **SHDI:** Shannon’s diversity index for land covers. **BAG:** El Bagre. **NEC:** Nechí. **CAU:** Caucasia.

In general, all the localities presented a fragmented landscape structure. La Lucha-BAG and Villa Grande-BAG showed the highest number and density of patches, which suggests that they have undergone highly active anthropization processes. La Lucha-BAG presented the highest MSIZ value, which underlines the high connectivity of its predominant cover. In contrast, Puerto Triana-CAU showed the lowest MSIZ (Table 3).

**Fig 3:**
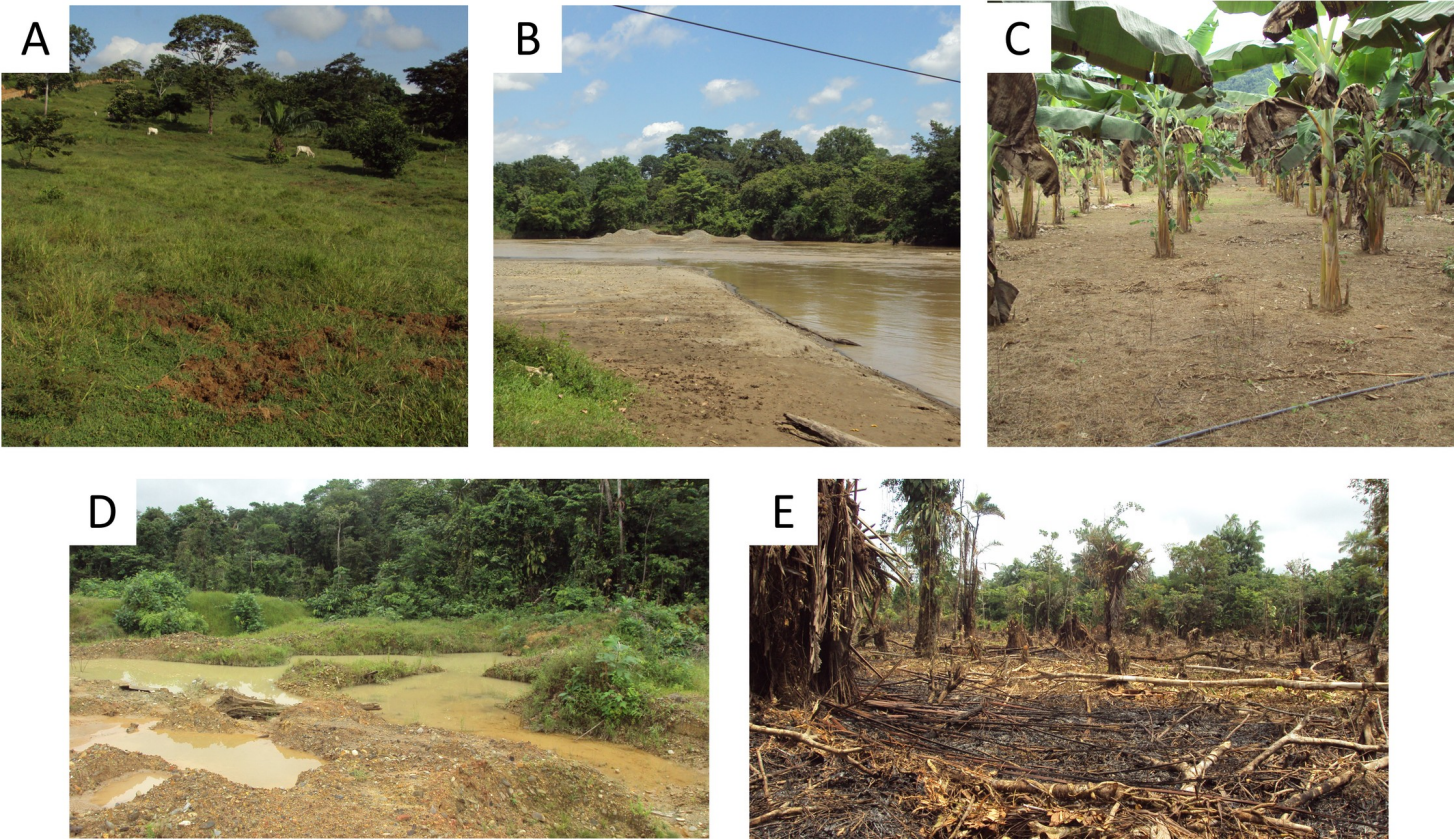
Representative land covers in the localities. Photographs representing some of the land covers in the localities of the Bajo Cauca region. (A) Grass intended for livestock. (B) Waterbody exploited for open-pit gold mining. (C) Crops. (D) Bare soil and wetland generated for open-pit mining. (E) Bare soil patch generated by deforestation (Photographs are a product of this work).

Field observations and orthophotograph classification allowed the definition of the main land uses. Forest cover resulted from natural forest succession, grasslands are used for cattle grazing (Fig 3A); also, various types of water bodies, including rivers, streams and natural or artificial ponds are often exploited for open-pit mining practices (Fig 3B). The crop cover was detected Villa Grande-BAG and Puerto Triana-CAU localities (Fig 3C). In El Bagre localities, bare soil patches are generated by deforestation, for open-pit mining or house construction (Fig 3D). In Caucasia and Nechí localities, bare soils and artificial wetlands resulted from mining activities (Fig 3E).

### *Anopheles* community and landscape features

**Fig 4:**
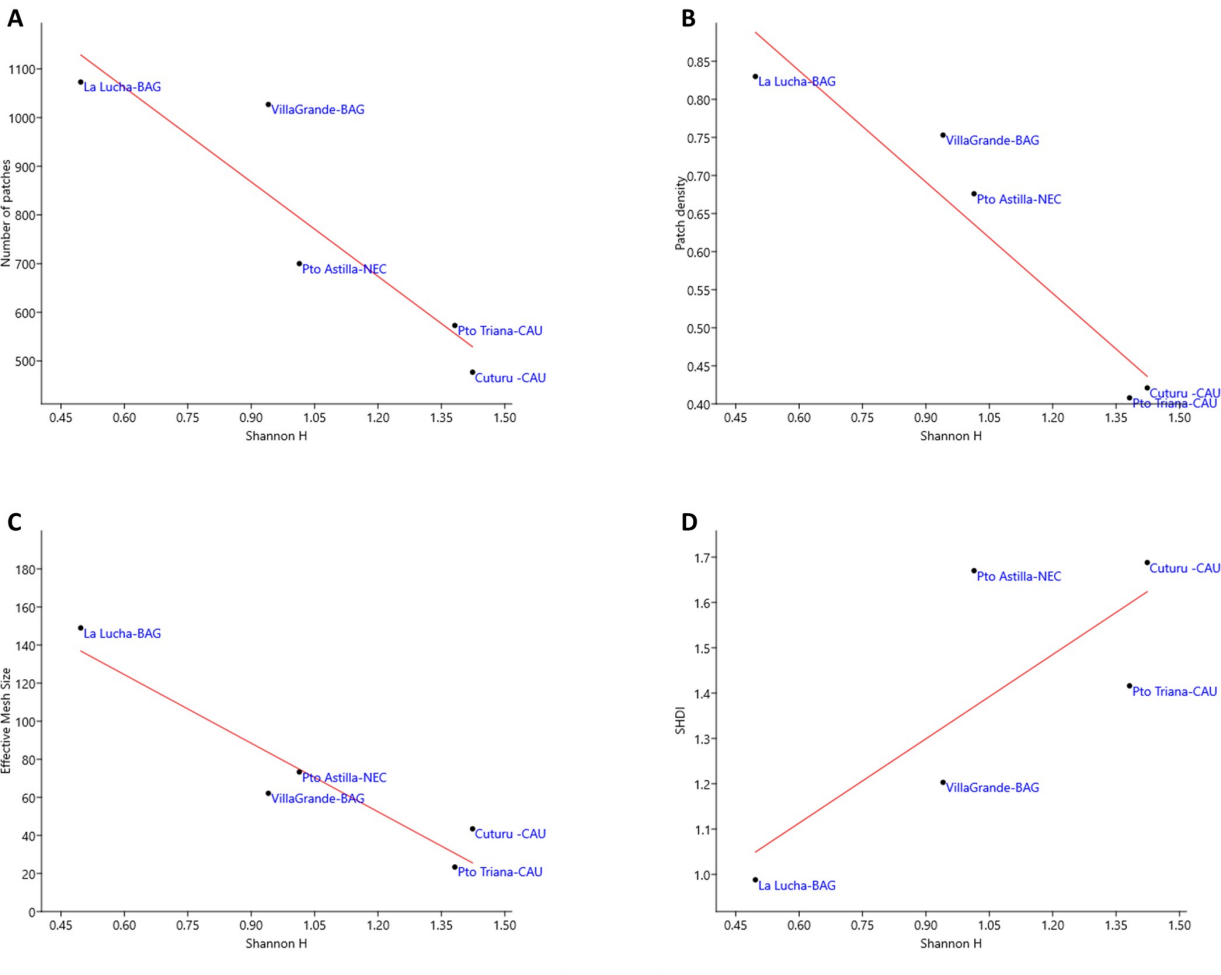
Relationship between *Anopheles* diversity (Shannon index) with landscape diversity and landscape fragmentation indices as estimated by simple linear regression analysis. *Anopheles* diversity with (A) number of patches, (B) patch density, (C) effective mesh size and (D) landscape diversity (SHDI).

The simple linear regression models showed a significant negative relationship for *Anopheles* diversity with the number of patches (r = - 0.91; r^2^ = 0.83; *p<*0.05) (Fig 4A) and with patch density (r = - 0.95; r^2^ = 0.91; *p<* 0.05) (Fig 4B); also, there was a significant negative relationship between *Anopheles* diversity and the effective mesh size (r = - 0.94; r^2^ = 0.89; *p<* 0.05) (Fig 4C). No significant relationship was found between *Anopheles* diversity and landscape diversity (r = 0.77; r^2^ = 0.59; *p>*0.05) (Fig 4D).

**Fig 5:**
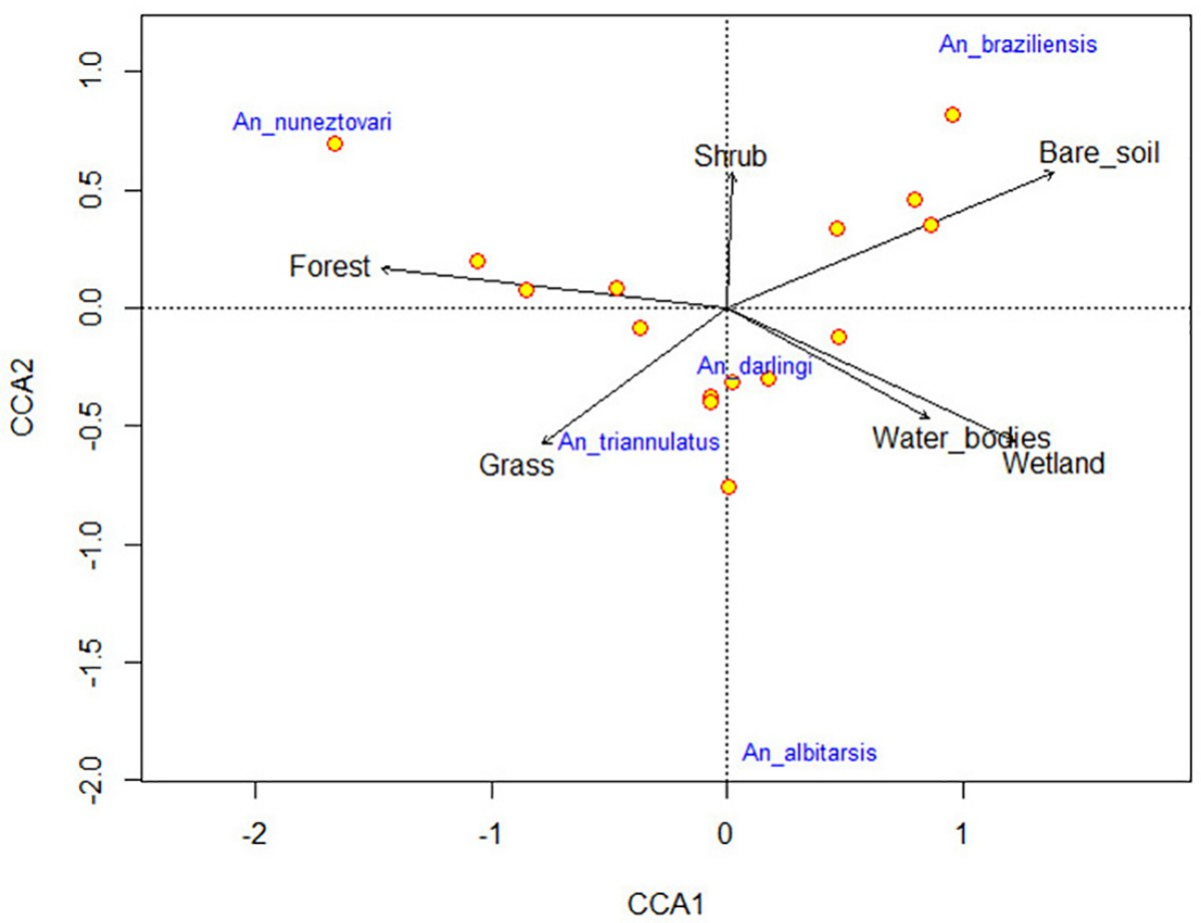
Relationship betwee*n Anopheles* species abundance and land cover as determined by Canonical Correspondence Analysis (CCA). In the figure, the yellow dots represent the sampling sites, the vectors (arrows) pointing in an increasing direction towards the land covers explain the abundance of particular species. The distance and position of species and vectors indicate the relationship between a land cover and the species abundance.

The CCA considered four canonical axes; the first two explained 64% of the data set variance (0.493 and 0.144, respectively). According to the permutation test applied to the CCA, the model and the first two canonical axes explained a higher variance of data than the expected by chance; the results of these tests were statistically significant (*p*<0.05). According to the CCA analysis, a higher abundance of *An*. *nuneztovari* was related to forest cover and to a lesser extent with shrub. *Anopheles darlingi* was located in the center of the canonical axis, showing a slight tendency of greater abundance related with wetland, water body and grass covers. While, *An*. *albitarsis* s.l. showed a tendency of being more abundant in landscapes composed by wetland and water body covers, and slightly with the grass cover. The abundance of *An*. *braziliensis* was strongly related to landscapes mainly composed of bare soil and showed a mid-to-low relationship with shrub covers. Finally, *An*. *triannulatus* s.l. was found in higher abundance in landscapes consisting mainly of grass cover (Fig 5).

## Discussion

In this study, 10 *Anopheles* species were identified in five localities of the Bajo Cauca region; this number corresponds to approximately 20% of the species reported for the entire country [35]. Of relevance, the Colombian primary malaria vectors *An*. *darlingi* and *An*. *nuneztovari* were present in all localities, although, varied in abundance. Of notice, these vectors were previously detected infected with *Plasmodium vivax* in various localities of El Bagre and constituted the most abundant species [31]; therefore, the present findings suggest that these two vector species are contributing to maintain malaria transmission in these localities of the Bajo Cauca region.

Regarding other *Anopheles* species detected, *An*. *braziliensis* was found in all localities except in La Lucha-BAG. It was the most abundant species in two localities, Puerto Astilla-NEC and Cuturú-CAU, mainly collected by HLC. In a previous work conducted more than ten years ago, this species was the second most abundant in localities of El Bagre and the most abundant in the neighbor municipality Zaragoza [30], which indicates that in this region there is an adequate environment for *An*. *braziliensis*. Interestingly, this species is considered zoophilic [52]; however, its capture in human landing catches shows a remarkable anthropophilic tendency, suggesting plasticity or adaptability in host selection for blood feeding [53]. Furthermore, *An*. *braziliensis* has been found infected with *Plasmodium* sp. in Brazil [54]; thus, it is essential to assess its role in malaria transmission in the Bajo Cauca region.

The species accumulation curve indicated that the sampling effort was adequate to collect the *Anopheles* species present in the Bajo Cauca region (S1 Fig). At the locality level, the curves appear close to reaching the asymptotic point; furthermore, the Chao-1 index showed that the number of expected species matched the number of observed species for each locality, except for La Lucha-BAG (Table 2), supporting the assumption of an adequate sampling. The low diversity detected for the *Anopheles* community (Shannon index < 2) (Table 2), is similar to the low diversity generally present in the *Culicidae* communities [55–58], which is frequently attributed to the sampling methods for mosquito collection [52, 59]. The methodology used in this work (human landing catches and resting in livestock corrals), was mainly directed to the capture anopheline mosquitoes that are attracted to humans and cattle, and could have contributed to detecting a lower anopheline diversity.

The simple linear regression models showed a significant negative correlation between *Anopheles* diversity and the effective mesh size, number and density of patches (Fig 4). This result indicates that highly fragmented landscapes, even conserving the connectivity of their matrix, tend to have a lower *Anopheles* species diversity. This type of landscape corresponds for example, to a forest exploited for lumber extraction where selective logging causes fragmentation of the forest matrix [60, 61]. This phenomenon was observed in La Lucha-BAG and Villa Grande-BAG, localities with the lowest *Anopheles* species diversity. Conversely, anthropic activities in forestall environments may allow exposition to solar beams and the formation of larval habitats that can be used by *Anopheles* species novel to the sites [23, 62], particularly, those attracted to humans and possibly, of greater epidemiological importance. These may be occurring in Puerto Astilla-NEC, Cuturú-CAU, and Puerto Triana-CAU, where there was a reduction in the forest matrix and higher *Anopheles* richness and diversity. These localities evidenced a high degree of forest disturbance and considerable presence of water bodies and wetlands; covers that generate larval habitats and may be exploited by a variety of species [29, 63]. As suggested in other studies, anthropically intervened landscapes usually present higher culicid diversity than wild environments [55, 64–66]; also, landscape modifications can increase invertebrate species proliferation [67].

There was not significant statistical relationship between *Anopheles* diversity and landscape diversity. Even though, a slight tendency for higher *Anopheles* diversity was observed in localities with the highest landscape diversity (Fig 4D). The landscape diversity index (DSHI) considers the number of land cover types and a proportional distribution among these covers. Then, a greater number of coverages proportionally distributed, suppose a higher heterogeneity, which would favor the presence of more varied habitat types that may be occupied by a higher number of species [66]. The lowest *Anopheles* diversity was registered in the locality La Lucha-BAG, where *An*. *nuneztovari* showed a high dominance (Dominance D = 0.761). This species is recognized for its ability to colonize anthropically impacted areas [31, 68, 69], like the high human-impacted forests present in this BAG locality. The CCA analysis showed a significant relationship indicating a strong association of higher abundance of *An*. *nuneztovari* with forest cover and slighter with shrub cover (Fig 5). Accordingly, in BAG localities, a transition from forest to shrub cover was observed (Fig 2).

Even though, in the CCA analysis, *An*. *darlingi* was located in the center of the canonical axis, increased abundance of this specie is slightly related to wetlands, grass and water body covers (Fig 5). These land covers are related to open-pit mining and livestock activities that take place in the region and seem to influence the presence of *An*. *darlingi*. Similarly, in a previous study, this species was dominant in another BAG locality (La Capilla), where open-pit mining and livestock were the main economic activities [31]. Comparable to *An*. *darlingi*, *An*. *albitarsis* s.l. showed a tendency of being more abundant in landscapes composed by wetland, water body and grass covers (Fig 5). Reports on *An*. *albitarsis* s.l. from Paraná State in Brazil and Province of Chaco in Argentina indicate that the specimens were mainly found in grasslands, various types of water bodies and areas under frequent flooding [70–72]. In the present study, *An*. *albitarsis* s.l. was found in landscapes with wetlands generated by artificially flooded areas used for open-pit gold mining in Cuturú-CAU, Puerto Triana-CAU, and Puerto Astilla-NEC (Fig 2). Considering that *An*. *albitarsis* s.l. belongs to a species complex composed of vector and non-vector species [73, 74], for control intervention purposes, it is essential to precisely define the environmental factors that determine the presence of specific species.

*Anopheles braziliensis* showed a strong tendency for higher abundance in landscapes with predominant bare soil and lower relationship with shrub covers. In accordance with this results, there are reports of *An*. *braziliensis* in areas deprived of forest coverage and in breeding sites exposed to sunlight [75–77]. The bare soil cover in Puerto Astilla-NEC and Cuturú-CAU localities appears as a result of mining exploitation and in these localities *An*. *braziliensis* was the most abundant species. In a previous study performed in Zaragoza, a municipality in the same region, larvae of this species were found in inundated mining excavations [30]; these observations suggest that this species is strongly related to the mining activity that takes place in the region. Furthermore, in this study, *An*. *braziliensis* was not related to the grass cover. These results contrast with those of studies carried out in Sifontes, Venezuela and Porto Velho-Rio Branco, Brazil, which relate this species to pasture areas [75, 76]. Although, many could be the reasons for the difference in results, one may be genetic variation between *An*. *braziliensis* populations separated by the Andes; it has been shown that this mountain chain acts as a geographic barrier for populations of *Anopheles* [32, 33, 78].

*Anopheles triannulatus* s.l. showed a high relationship with grass coverage. The highest abundance of this species occurred in Puerto Triana-CAU, where grass coverage used for cattle grazing was the predominant land cover type (Table 3). This species has been recognized for its zoophilic tendency [30, 53, 79], which agrees with the higher proportion of *An*. *triannulatus* s.l. specimens collected resting in corrals as compared to those attracted to humans in Puerto Triana-CAU (Table 1). Greater availability of cattle as a blood-meal source seems to favor its presence in landscapes with abundant grazing areas. Although, *An*. *triannulatus* s.l. is a complex of at least three species which differ in vectorial capacity [79, 80], it is essential to identify the ecological requirements of each species in the complex for the design of appropriate vector control interventions.

## Conclusion

In the localities of the malaria endemic Bajo Cauca region, a strong association was found between *Anopheles* species abundance and land covers modified by anthropic activities, particularly, those derived from open-pit gold mining, livestock, deforestation and logging. These activities generate changes in the landscape structure that affect the spatial distribution of epidemiologically important species in the region. Furthermore, *Anopheles* species diversity and abundance were influenced by land cover composition and landscape fragmentation; the loss in the forest matrix changes species composition which may modify malaria distribution. Studies on the relationship between *Anopheles* and landscape structure are useful to predict scenarios that favor species presence. This information is useful for the implementation of vector control interventions directed to the conservation of landscapes favorable for malaria prevention.

## Supporting information

S1 Fig Species accumulation curves. (A) Bajo Cauca region. (B) By locality.
(TIFF)

S1 Table Land covers description. Land cover types were label according to categories of national land cover legends by the Instituto de Hidrología, Meteorología y Estudios Ambientales of Colombia (IDEAM).
(DOCX)

S1 Data Statistical results of the linear regression and CCA. (XLSX)
