# Supplementary figures and images for: Effect of land cover and landscape fragmentation on anopheline mosquito abundance and diversity in an important Colombian malaria endemic region
Source: PLoS One. 2020 Oct 15;15(10):e0240207. doi: 10.1371/journal.pone.0240207 (PMC7561141; doi:10.1371/journal.pone.0240207)

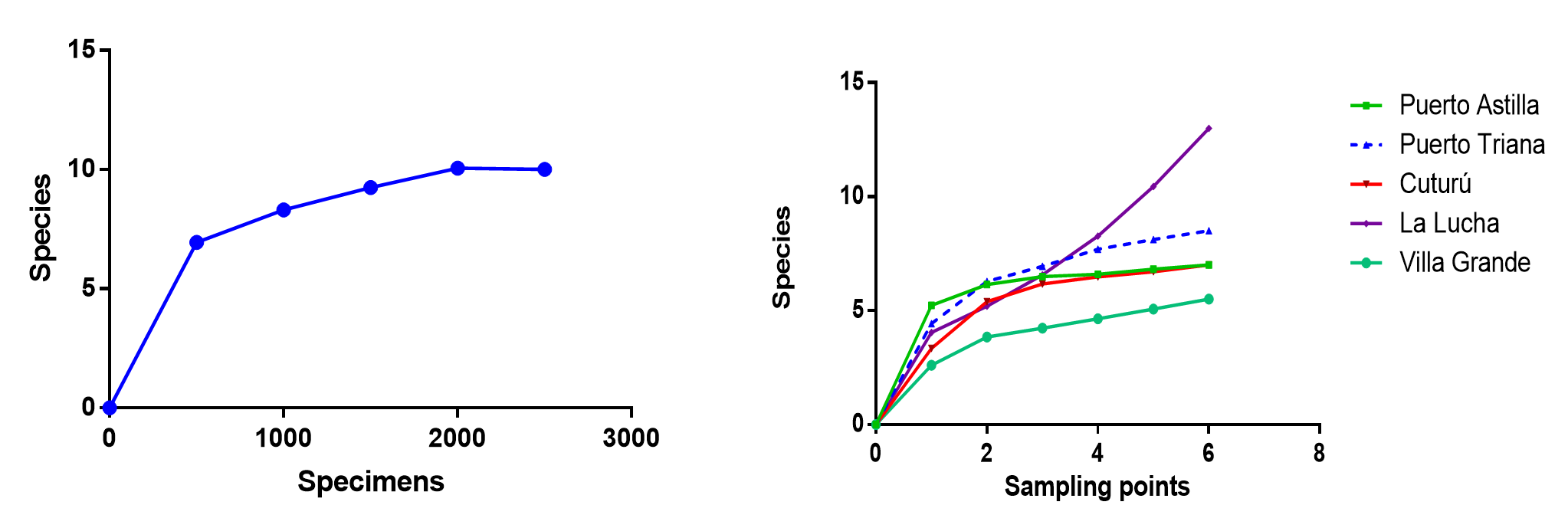

Supplement: S1 Fig — (A) Bajo Cauca region. (B) By locality. (TIFF) [file pone.0240207.s001.tiff]
