# Supplementary material for: Effect of land cover and landscape fragmentation on anopheline mosquito abundance and diversity in an important Colombian malaria endemic region
Source: PLoS One. 2020 Oct 15;15(10):e0240207. doi: 10.1371/journal.pone.0240207 (PMC7561141; doi:10.1371/journal.pone.0240207)
Supplement: S1 Table — Land cover types were label according to categories of national land cover legends by the Instituto de Hidrología, Meteorología y Estudios Ambientales of Colombia (IDEAM). (DOCX) [file pone.0240207.s002.docx]

S1 Table. Description of land covers.

| **Land cover** | **Description*** |
| --- | --- |
| **Forest** | Areas constituted by arboreal elements with tree tops defined or barely defined |
| **Shrub** | Mainly shrubby vegetation with irregular dossal and presence of shrubs, palms and low vegetation. |
| **Grass** | Land occupied by neat grass covering 70% or more of its extension |
| **Crop** | Terrains mainly dedicated to food, fiber and raw material production |
| **Bare soil** | Territory with scarce or no vegetation composed by burned and bare soils likewise sandy covers and rocky outcrop |
| **Water bodie** | Permanent, intermittent and seasonal water conformed by lakes, lagoons, water tanks, natural or artificial freshwater ponds, dams, water flows like rivers and waterways. |
| **Wetland** | Waterlogging zones and swamps in which the phreatic level it’s at ground level |

* Land cover types were label according to categories of national land cover legends by the Instituto de Hidrología, Meteorología y Estudios Ambientales de Colombia (IDEAM, 2010).
